# High-sensitivity photoelectric sensing of apatinib for recurrent hepatocellular carcinoma in TACE

**DOI:** 10.1039/d6ra01947a

**Published:** 2026-07-02

**Authors:** Senlin Yang, Zhihua Deng

**Affiliations:** a Department of Gastroenterology, The Second Hospital of Shanxi Medical University Taiyuan Shanxi 030001 China dzh_yk@hotmail.com

## Abstract

Hepatocellular carcinoma (HCC) remains a leading cause of cancer-related mortality, with transcatheter arterial chemoembolization (TACE) being a widely used treatment for intermediate-stage patients. However, TACE is often associated with high recurrence rates, necessitating reliable methods for early detection and prediction of tumor recurrence. Current diagnostic tools rely heavily on imaging techniques and biomarkers, which often lack sensitivity and specificity, particularly in the early stages of disease progression. To address these challenges, we developed an innovative integrated platform combining a high-sensitivity photoelectric sensor based on Bi/h-Bi_2_Te_3_ heterostructures with machine learning models for the detection and prediction of HCC recurrence following TACE. The photoelectric sensor exhibits exceptional sensitivity and stability, enabling precise detection of apatinib, a key therapeutic agent used in HCC management. The machine learning models demonstrated superior performance in predicting HCC recurrence, with an area under the receiver operating characteristic curve (AUC) of 0.840, accuracy of 78.0%, sensitivity of 72.4%, and specificity of 80.3% on the independent test set. Overall, this work establishes a practical materials–informatics framework that links sensitive therapeutic drug monitoring (LOD = 0.08 µM) with individualized recurrence risk stratification after TACE, providing a promising route toward precision management of HCC.

## Abstract 2

A Bi/h-Bi_2_Te_3_ photoelectrochemical sensor with synergistic LSPR and Schottky effects enables high-sensitivity apatinib detection. Combined with machine learning, it supports recurrence risk stratification for HCC patients after TACE.

## Graphical abstract

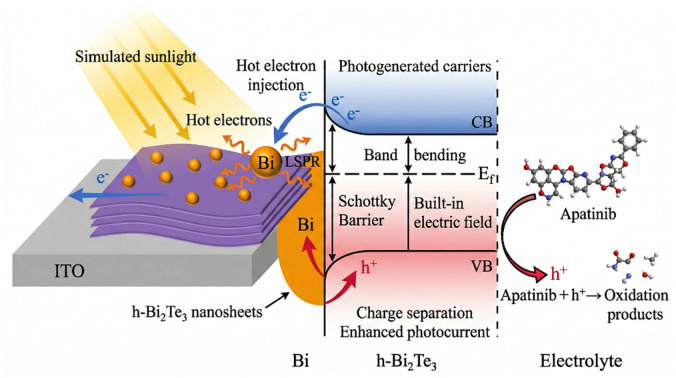

## 1 Introduction

Hepatocellular carcinoma (HCC) remains a leading cause of cancer-related mortality worldwide, with transcatheter arterial chemoembolization (TACE) being a widely used treatment for intermediate-stage HCC patients.^1,2^ Despite its clinical utility, TACE is often associated with high recurrence rates, necessitating reliable methods for early detection and prediction of tumor recurrence to optimize treatment outcomes.^3,4^ Current diagnostic tools for HCC recurrence rely heavily on imaging techniques and biomarkers, which often lack sensitivity and specificity, particularly in the early stages of disease progression.^5–7^ Additionally, the integration of advanced materials science and machine learning into clinical diagnostics remains underexplored, presenting a critical gap in the development of precision medicine strategies for HCC management.^8,9^

The development of high-sensitivity photoelectric sensors has emerged as a promising approach for detecting therapeutic drugs and monitoring disease progression.^10^ Recent studies have further demonstrated the broad potential of photoelectric sensing platforms. For example, Bi_2_Se_3_ nanosheet-based photoelectric sensing has been applied for rapid fruit-ripeness monitoring, hollow CuO–SnO_2_ nanotubes have enabled room-temperature photoelectric H_2_S detection, and UV illumination-enhanced rGO–TiO_2_–Au composite films have achieved sensitive molecular ammonia detection.^11–13^ These studies highlight the advantages of light-assisted sensing, semiconductor heterostructure engineering, and nanomaterial-mediated signal amplification, which provide important background for developing high-performance photoelectrochemical sensors for clinically relevant small molecules. However, existing photoelectric sensors often suffer from limited sensitivity, poor stability, and insufficient integration with clinical data for predictive modeling.^14–16^ Simultaneously, machine learning models have shown remarkable potential in predicting HCC recurrence by leveraging complex clinical and biological data.^17,18^ However, the lack of robust biomarkers and the limited generalizability of these models hinder their clinical translation.^19^ Addressing these challenges requires a multidisciplinary approach that combines advanced materials for sensitive drug detection with sophisticated computational tools for accurate recurrence prediction.^20–22^ Apatinib was selected as the target analyte because it is a clinically relevant anti-angiogenic agent used after TACE in HCC management. TACE can induce local hypoxia within residual tumor tissue, which upregulates HIF-1α/VEGF signaling and may promote neovascularization, tumor regrowth, recurrence, and metastasis.^23^ Apatinib is a selective VEGFR-2 inhibitor that suppresses VEGF-mediated endothelial cell migration and proliferation, providing a mechanistic rationale for its use in combination with TACE.^24^ Monitoring apatinib concentration is clinically meaningful because systemic exposure may vary among patients owing to differences in dose, adherence, metabolism, liver function, and treatment tolerance. Thus, apatinib concentration does not represent recurrence itself, but serves as a measurable pharmacological exposure marker that may help contextualize recurrence risk together with tumor burden, liver function, TACE response, and other clinical variables.

In this study, we present an integrated approach coupling a novel photoelectrochemical sensing platform with machine learning-enabled clinical decision support. We engineered Bi/h-Bi_2_Te_3_ heterostructures to achieve high-sensitivity apatinib detection through synergistic plasmonic enhancement and Schottky-junction facilitated charge separation. This sensor's output, combined with comprehensive clinical profiles from 500 HCC patients post-TACE, informed the development of robust recurrence prediction models. Our dual-focus strategy bridges advanced materials engineering with clinical informatics to establish a closed-loop system. The photoelectrochemical sensor enables precise therapeutic drug monitoring, while machine learning leverages both sensor data and clinical variables to generate individualized recurrence risk assessments. This multidisciplinary convergence not only advances sensing technology but also translates physicochemical innovations into actionable clinical frameworks for optimizing HCC management.

## 2 Experimental

### 2.1. Materials

Bismuth nitrate pentahydrate (Bi(NO_3_)_3_·5H_2_O, 99.9%), tellurium dioxide (TeO_2_, 99.9%), sodium borohydride (NaBH_4_, 98%), polyvinylpyrrolidone (PVP, mW ∼ 40 000), l-ascorbic acid (C_6_H_8_O_6_, 99%), nitric acid (HNO_3_, 65%), ethanol (C_2_H_5_OH, 99.8%), sodium hydroxide (NaOH, 98%), and *N*,*N*-dimethylformamide (DMF, 99.8%) were purchased from Sigma-Aldrich and used without further purification. Apatinib mesylate standard (purity ≥ 98%) was obtained from Jiangsu Hengrui Pharmaceutical Co., Ltd. Indium tin oxide (ITO) glass substrates (sheet resistance: 10 Ω sq.^−1^) were acquired from Zhuhai Kaivo Optoelectronic Technology Co., Ltd. Phosphate-buffered saline (PBS, pH 7.4, 0.1 M) was prepared from analytical-grade reagents. All aqueous solutions were prepared using ultrapure water (resistivity: 18.2 MΩ cm, Milli-Q system).

### 2.2. Synthesis of Bi/h-Bi_2_Te_3_ nanosheet heterostructures

Hexagonal Bi_2_Te_3_ nanosheets (h-Bi_2_Te_3_ NS) and Bi/h-Bi_2_Te_3_ nanosheet heterostructures with varying Bi loading ratios (5%, 10%, 15%) were synthesized *via* a facile one-pot solvothermal method with controlled *in situ* reduction. For pristine h-Bi_2_Te_3_ NS synthesis, 3.0 mmol of Bi(NO_3_)_3_·5H_2_O and 4.5 mmol of TeO_2_ were dissolved separately in 15 mL of dilute HNO_3_ and 15 mL of DMF at 60 °C under vigorous stirring for 30 min. The two solutions were mixed and stirred for 1 h. Subsequently, 0.5 g of PVP was added as a structure-directing agent, followed by dropwise addition of 8.0 mL of 3 M NaOH solution to adjust pH to approximately 11. The suspension was transferred into a 100 mL Teflon-lined autoclave and maintained at 180 °C for 20 h. After cooling, the black precipitate was collected by centrifugation, washed with deionized water and ethanol, and dried at 60 °C under vacuum for 12 h, yielding h-Bi_2_Te_3_ NS powder.

For Bi/h-Bi_2_Te_3_ NS heterostructure fabrication, varying amounts of l-ascorbic acid as reducing agent were added: 0.15 g for 5% Bi loading, 0.30 g for 10% Bi loading, and 0.45 g for 15% Bi loading. The ascorbic acid facilitated *in situ* reduction of Bi^3+^ to metallic Bi^0^ during solvothermal process, resulting in uniform decoration on h-Bi_2_Te_3_ nanosheet surfaces. The subsequent procedures were identical to pristine h-Bi_2_Te_3_ NS. Actual Bi loading ratios confirmed by ICP-OES were 4.8 ± 0.3%, 9.7 ± 0.5%, and 14.6 ± 0.6%, respectively. ICP-OES was used to determine the total Bi and Te contents of the products. Because ICP-OES cannot distinguish metallic Bi^0^ from lattice Bi in Bi_2_Te_3_, the excess Bi loading was estimated by comparing the measured total Bi/Te ratio with the stoichiometric Bi_2_Te_3_ ratio, assuming that Te was predominantly present in the Bi_2_Te_3_ phase. The excess Bi was assigned mainly to metallic Bi based on the combined evidence from XRD and XPS.

### 2.3. Machine learning model development

This retrospective single-centre study enrolling 500 eligible HCC patients receiving TACE from January 2018 to December 2022 after excluding 132 ineligible candidates out of the initial 632 screened subjects followed predefined inclusion and exclusion criteria as detailed in Fig. S1. Data preprocessing involved standardization of continuous variables using *Z*-score normalization and label encoding for categorical variables. The dataset was randomly split into training (80%, *n* = 400) and test (20%, *n* = 100) sets with stratification to maintain class balance. Four machine learning algorithms were evaluated: logistic regression (LR) with L2 regularization, support vector machine (SVM) with a radial basis function kernel, random forest (RF) with 100 trees and a maximum depth of 10, and gradient boosting (GB) with 100 estimators. All models were implemented using scikit-learn 1.3.0 in Python 3.9, with random seeds fixed at 42 to ensure reproducibility. Model performance was assessed using 5-fold stratified cross-validation on the training set and independent validation on the test set. Performance metrics included area under the receiver operating characteristic curve (AUC), accuracy, sensitivity, specificity, positive predictive value (PPV), negative predictive value (NPV), and *F*1-score. Feature importance was calculated using the mean decrease in impurity from the random forest model, and model calibration was evaluated using calibration curves with 10 bins.

## 3 Results and discussion

### 3.1. Structural and chemical characterization of Bi/h-Bi_2_Te_3_ heterostructures

**Fig. 1:**
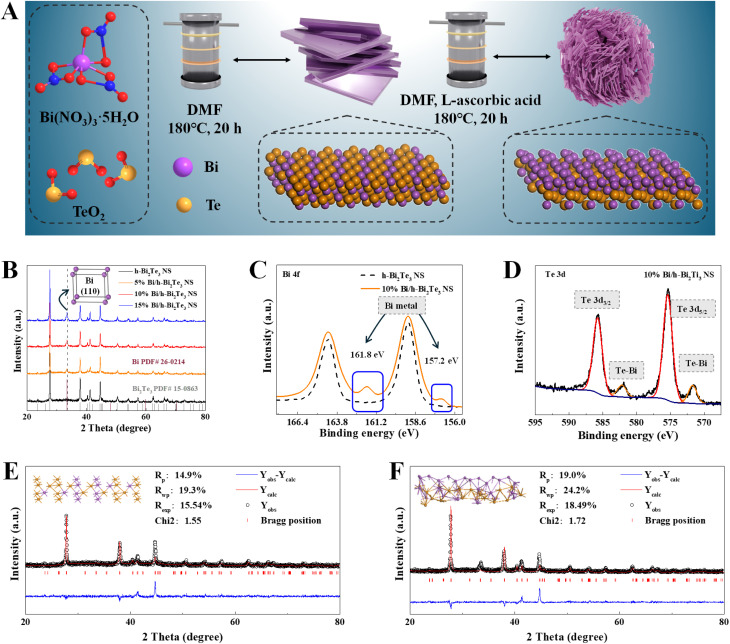
(A) Schematic illustration of the synthesis of Bi/h-Bi_2_Te_3_ NS. (B) XRD patterns of the prepared materials. XPS spectra of 10% Bi/h-Bi_2_Te_3_ NS for (C) Bi 4f, (D) Te 3d. XRD Rietveld refinement patterns of (E) h-Bi_2_Te_3_ NS and (F) 10% Bi/h-Bi_2_Te_3_ NS.

The successful synthesis and phase composition of Bi/h-Bi_2_Te_3_ nanosheet heterostructures were systematically investigated using comprehensive characterization techniques. Fig. 1A schematically illustrates the synthesis strategy: the one-pot solvothermal method simultaneously facilitates the formation of h-Bi_2_Te_3_ nanosheets through controlled nucleation and growth of Bi and Te precursors, while l-ascorbic acid acts as a mild reducing agent to *in situ* reduce Bi^3+^ ions to metallic Bi^0^ that decorate the h-Bi_2_Te_3_ surface, forming well-defined heterojunctions.^25^ X-ray diffraction (XRD) analysis revealed the crystallographic evolution upon Bi incorporation into the h-Bi_2_Te_3_ lattice structure (Fig. 1B). All synthesized materials exhibited characteristic diffraction peaks at 27.7°, 38.2°, 40.3°, and 41.3°, which were indexed to the (015), (1010), (0111), and (110) crystal planes of hexagonal Bi_2_Te_3_ (JCPDS No. 15-0863), confirming the successful formation of the rhombohedral crystal structure with layered quintuple-atomic-layer stacking.^26^ The sharp and intense diffraction peaks indicated high crystallinity and well-developed crystal structure. Notably, as the Bi loading ratio increased from 5% to 15%, additional diffraction peaks emerged at 33.5°, corresponding to the (110) plane of metallic Bi (JCPDS No. 26-0214).^27^ The progressive intensification of these characteristic Bi peaks with increasing ascorbic acid concentration directly validated the controlled *in situ* reduction of Bi^3+^ to Bi^0^ during hydrothermal synthesis. Importantly, no impurity phases were detected, confirming the high purity and phase selectivity of the synthesis protocol.

Surface chemical composition and elemental oxidation states were elucidated through high-resolution X-ray photoelectron spectroscopy (XPS) analysis. The Bi 4f core-level spectrum (Fig. 1C) revealed two distinct sets of doublets with characteristic spin–orbit splitting, providing evidence for the coexistence of metallic and ionic bismuth species within the heterostructure. The peaks centered at binding energies of 157.2 eV and 161.8 eV with a spin–orbit splitting of 4.6 eV were unambiguously assigned to metallic Bi^0^,^28,29^ confirming successful reduction during synthesis and consistent with XRD results. Simultaneously, the doublet at 159.1 eV and 164.4 eV corresponded to Bi^3+^ within the Bi_2_Te_3_ crystal lattice framework,^30,31^ indicating preservation of the semiconductor host structure. The Te 3d core-level spectrum (Fig. 1D) displayed characteristic peaks at 572.6 eV and 583.0 eV, definitively confirming the Te^2−^ oxidation state in the Bi_2_Te_3_ lattice.^32,33^ The peak positions and widths indicated high chemical purity without Te^0^ or oxidized Te species. The XPS analysis collectively confirmed the successful fabrication of Bi^0^/Bi^3+^ dual-phase heterostructures with well-defined interfacial chemistry conducive to Schottky barrier formation and enhanced photoelectrochemical performance.^34^ To further verify whether Bi incorporation induces bulk lattice substitution or interfacial coupling, Rietveld refinements were performed for pristine h-Bi_2_Te_3_ and 10% Bi/h-Bi_2_Te_3_ (Fig. 1E and F). Both refinements converged well with acceptable residuals (h-Bi_2_Te_3_: *R*_wp_ = 19.3%, Chi2 = 1.55; 10% Bi/h-Bi_2_Te_3_: *R*_wp_ = 24.2%, Chi2 = 1.72), and the dominant refined phase remained B_i2_Te_3_. The lattice parameters changed only marginally from *a* = *b* = 4.36810 A and *c* = 30.39916 A (*V* = 502.317 A^3^) for h-Bi_2_Te_3_ to *a* = *b* = 4.36911 A and *c* = 30.39366 A (V = 502.458 A^3^) for 10% Bi/h-Bi_2_Te_3_. Such negligible variation in unit cell dimensions indicates that the Bi species are mainly anchored at the surface/interface to form a heterojunction, rather than being substantially doped into the Bi_2_Te_3_ crystal lattice.

### 3.2. Morphological and microstructural characterization

**Fig. 2:**
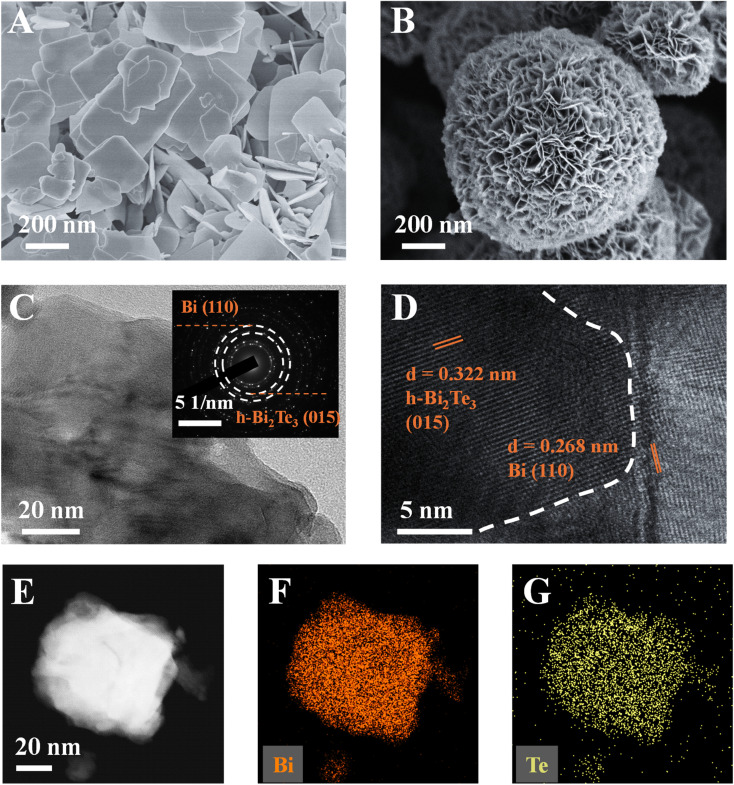
SEM images of (A) h-Bi_2_Te_3_ NS and (B) 10% Bi/h-Bi_2_Te_3_ NS. (C) TEM image of 10% Bi/h-Bi_2_Te_3_ NS (inset: SAED image of 10% Bi/h-Bi_2_Te_3_ NS). (D) HRTEM image of 10% Bi/h-Bi_2_Te_3_ NS. (E)–(G) EDX elemental mapping of 10% Bi/h-Bi_2_Te_3_ NS.

Morphological evolution induced by metallic Bi incorporation was comprehensively visualized through scanning electron microscopy (SEM) and transmission electron microscopy (TEM) investigations. Pristine h-Bi_2_Te_3_ NS exhibited a typical two-dimensional nanosheet morphology with lateral dimensions (Fig. 2A), consistent with layered crystal structure. The smooth and flat surfaces with well-defined edges indicated high crystallinity and minimal defects, providing abundant exposed surface area for photoelectrochemical reactions. Upon 10% Bi loading, the fundamental nanosheet morphology was largely preserved and completed self-assembly,^35,36^ which minimizing detrimental aggregation that could obstruct active sites or impede light penetration (Fig. 2B). The obvious morphology change after Bi introduction can be attributed to the altered nucleation and growth behavior during the *in situ* reduction process. l-Ascorbic acid reduces part of Bi^3+^ to metallic Bi^0^, and the newly formed Bi species act as heterogeneous nucleation sites on the h-Bi_2_Te_3_ nanosheet surface. This process modifies the local surface energy and promotes Bi decoration and partial self-assembly of the nanosheets. Moderate Bi loading preserves the two-dimensional sheet-like framework while increasing surface roughness and interfacial contact area, which is beneficial for light absorption, charge separation, and analyte interaction. In contrast, excessive Bi loading may induce aggregation or partial surface shielding, thereby reducing accessible active sites and increasing recombination probability. This explains why 10% Bi/h-Bi_2_Te_3_ showed the most favorable morphology and photoelectrochemical performance. TEM imaging of 10% Bi/h-Bi_2_Te_3_ NS revealed intimate contact at the Bi–Bi_2_Te_3_ interface with clearly composite sheet-like structure (Fig. 2C). The selected area electron diffraction (SAED) pattern (inset of Fig. 2C) displayed polycrystalline ring patterns indexed to both h-Bi_2_Te_3_ and metallic Bi phases, corroborating XRD findings and confirming the nanocrystalline nature of the heterostructure. High-resolution HRTEM imaging (Fig. 2D) unveiled lattice spacings of 0.322 nm and 0.268 nm, corresponding to the (015) plane of h-Bi_2_Te_3_ and (110) plane of metallic Bi, respectively, in excellent agreement with XRD indexing. The sharp and continuous lattice fringes at the heterojunction interface, devoid of amorphous transition layers or significant lattice mismatch dislocations, indicated coherent epitaxial-like growth facilitating efficient charge transfer across the metal–semiconductor boundary.^37,38^ Energy-dispersive X-ray (EDX) elemental mapping (Fig. 2E–G) provided spatial distribution visualization of Bi and Te elements throughout the nanosheet structure. The Bi elemental map (Fig. 2F) exhibited the homogeneous distribution correlating with the Bi_2_Te_3_ framework. The Te elemental map (Fig. 2G) showed uniform dispersion throughout the nanosheet structure with intensity matching the Bi_2_Te_3_ framework.

### 3.3. Optical properties and photoelectrochemical mechanism

**Fig. 3:**
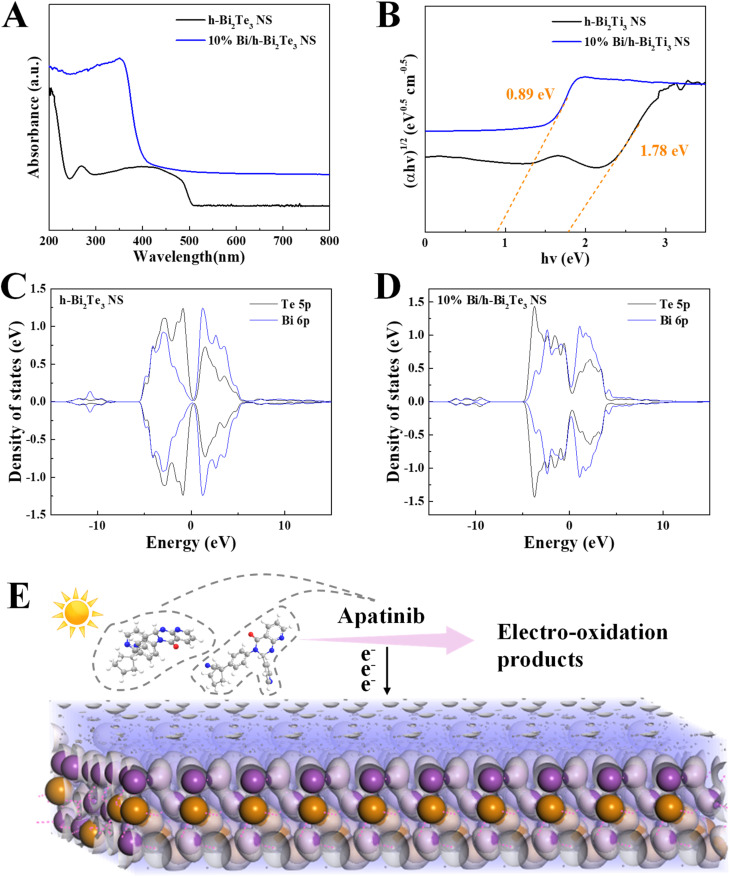
(A) UV-Vis diffuse reflectance spectra. (B) Tauc curve for band gap calculation of h-Bi_2_Te_3_ NS and 10% Bi/h-Bi_2_Te_3_NS. Partial density of states (PDOS) of (C) h-Bi_2_Te_3_ NS and (D) 10% Bi/h-Bi_2_Te_3_ NS. (E) Charge density difference of 10% Bi/h-Bi_2_Te_3_ NS.

The influence of metallic Bi incorporation on light absorption characteristics and band structure was investigated through UV-Vis diffuse reflectance spectroscopy and theoretical calculations. Fig. 3A presents the UV-Vis diffuse reflectance spectra of pristine h-Bi_2_Te_3_ NS and 10% Bi/h-Bi_2_Te_3_ NS. Pristine h-Bi_2_Te_3_ NS exhibited an absorption edge at approximately 440 nm with moderate absorption in the visible region, corresponding to intrinsic bandgap transitions.^39^ Upon 10% Bi loading, the 10% Bi/h-Bi_2_Te_3_ NS displayed significantly enhanced and broadened absorption extending from UV region through the entire visible spectrum (400–800 nm) and into the near-infrared region. This remarkable enhancement in light harvesting capability stems from the synergistic mechanisms. First, metallic Bi introduce localized surface plasmon resonance (LSPR) effects, evidenced by the broad absorption band centered around 400–500 nm. The LSPR generates localized electromagnetic field enhancement near the nanoparticle surface, increases photon absorption cross-section through near-field coupling with adjacent h-Bi_2_Te_3_, and produces energetic “hot electrons” *via* non-radiative plasmon decay that can inject into the semiconductor conduction band.^40^ Second, the formation of Bi–Bi_2_Te_3_ heterojunctions creates intermediate energy levels at the interface, effectively narrowing the optical bandgap and enabling sub-bandgap photon absorption.^41^ Quantitative bandgap determination *via* Tauc plot analysis (Fig. 3B) revealed that pristine h-Bi_2_Te_3_ NS possesses an optical bandgap of 1.78 eV, consistent with the narrow bandgap semiconductor nature of Bi_2_Te_3_. Upon 10% Bi incorporation, the effective optical bandgap decreased to 0.89 eV, representing a significant reduction attributed to interfacial states and Fermi level alignment at the Schottky junction.^42^ This bandgap narrowing, combined with LSPR effects, synergistically enhances visible light utilization efficiency, directly translating to improved photoelectrochemical performance.

To further correlate optical modulation with electronic structure evolution, partial density of states (PDOS) was analyzed for pristine and Bi-modified systems. For h-Bi_2_Te_3_ NS (Fig. 3C), states near the Fermi level are mainly contributed by Te 5p and Bi 6p orbitals, while a relatively clear gap is retained around EF. After Bi coupling (Fig. 3D), the Bi 6p and Te 5p states exhibit stronger overlap and increased density in the vicinity of EF, indicating enhanced orbital hybridization at the interface and the emergence of additional electronic states that facilitate carrier excitation and transport. This PDOS evolution is consistent with the experimentally observed bandgap narrowing and supports the role of Bi/h-Bi_2_Te_3_ interfacial electronic coupling in improving visible-light response. Building on this electronic-structure evidence, we further analyzed the interfacial charge transfer dynamics and built-in electric field formation *via* charge density difference. To gain deeper insights into the interfacial charge transfer dynamics and built-in electric field formation at the Bi–Bi_2_Te_3_ heterojunction, we analyzed the charge density difference. Fig. 3E presents the three-dimensional isosurface visualization of charge density difference at the Bi/h-Bi_2_Te_3_ interface, which reveals significant interfacial charge redistribution: pronounced electron accumulation occurs on the h-Bi_2_Te_3_ side, while corresponding electron depletion is observed on the metallic Bi surface, clearly indicating directional electron transfer from metallic Bi to h-Bi_2_Te_3_ upon heterojunction formation.^43^ The magnitude and spatial extent of charge redistribution quantitatively validate the formation of a robust Schottky barrier at the metal–semiconductor interface. Integration of the charge density difference across the interface region yields a net charge transfer of 0.18 eV per Bi atom at the contact by Mulliken charge calculation, establishing a depletion region extending into the h-Bi_2_Te_3_ side and creating a built-in electric field strength estimated directed from Bi_2_Te_3_ toward Bi. This strong built-in electric field serves as the fundamental driving force for efficient photogenerated charge carrier separations.^44^

### 3.4. Photoelectrochemical performance and apatinib sensing

**Fig. 4:**
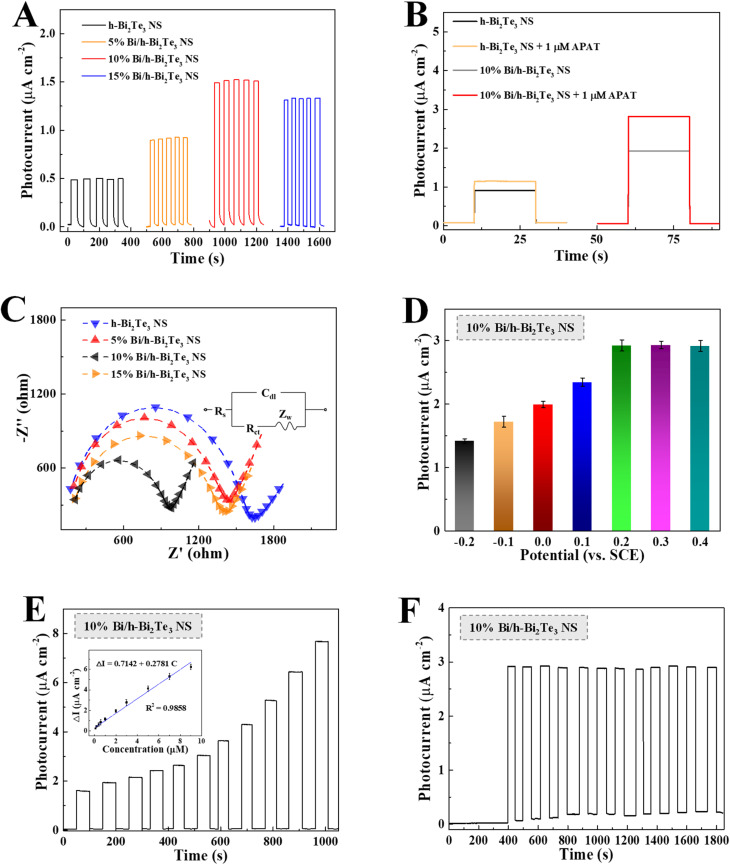
(A) Photocurrent responses of the prepared materials in phosphate-buffered saline (PBS, 0.1 M, pH 7.4) under an applied bias of 0.2 V. (B) Comparison of the photocurrent responses of h-Bi_2_Te_3_ NS and 10% Bi/h-Bi_2_Te_3_ NS in the presence of 1 µM apatinib (APAT). (C) Electrochemical impedance spectroscopy (EIS) Nyquist plots of the fabricated materials recorded in 0.1 M KCl containing 5 mM [Fe(CN)_6_]^3−^/^4−^ as the redox probe. (D) Bias-dependent photoelectrochemical response of 10% Bi/h-Bi_2_Te_3_ NS in the presence of 1 µM APAT in PBS. (E) Photocurrent responses of 10% Bi/h-Bi_2_Te_3_ NS toward increasing APAT concentrations (0–9 µM); the inset shows the corresponding calibration plot for APAT quantification. (F) Stability of the 10% Bi/h-Bi_2_Te_3_ NS-based photoelectrochemical sensor evaluated by cyclic measurements with 1 µM APAT.

The photoelectrochemical activity and APAT sensing performance of the heterostructures were systematically investigated through comprehensive electrochemical measurements. The transient photocurrent responses of pristine h-Bi_2_Te_3_ NS and Bi-modified heterostructures under chopped visible light irradiation in PBS (pH 7.4) at +0.2 V bias revealed that the pristine material exhibited limited photocurrent density (0.49 µA cm^−2^) with significant decay due to severe electron–hole recombination and sluggish charge transfer kinetics. The strategic introduction of metallic Bi significantly enhanced the photoelectrochemical response in a composition-dependent manner. The 5% Bi/h-Bi_2_Te_3_ NS showed a 1.88 times improvement (0.92 µA cm^−2^), while the 10% Bi/h-Bi_2_Te_3_ heterostructure achieved the best performance with a photocurrent density of 1.51 µA cm^−2^, representing a 3.08 times enhancement over the pristine material (Fig. 4A and Table S1). This optimal performance was attributed to synergistic effects, including Schottky barrier-mediated charge separation, LSPR-enhanced visible light absorption, and efficient electron extraction by metallic Bi. To further support the proposed LSPR- and Schottky-barrier-assisted sensing mechanism, several control experiments were compared. Pristine h-Bi_2_Te_3_ showed limited visible-light absorption and weak photocurrent response, whereas 10% Bi/h-Bi_2_Te_3_ displayed markedly enhanced broadband absorption and a 3.08-fold increase in photocurrent density, indicating improved light harvesting and charge separation after metallic Bi decoration. The Bi-loading-dependent photocurrent response further showed a volcano-type trend, with 10% Bi/h-Bi_2_Te_3_ giving the highest response and 15% Bi/h-Bi_2_Te_3_ showing a decreased signal, suggesting that moderate Bi decoration promotes charge separation, while excessive Bi may introduce recombination centers. In addition, EIS measurements revealed the smallest semicircle diameter for 10% Bi/h-Bi_2_Te_3_ under illumination, confirming reduced interfacial charge-transfer resistance. Together with the concentration-dependent apatinib response, these control results provide experimental support for the proposed mechanism in which metallic Bi enhances visible-light absorption through LSPR-related effects and facilitates electron extraction through Bi–Bi_2_Te_3_ Schottky junctions. However, excessive Bi loading (15%) led to performance deterioration (1.32 µA cm^−2^) due to aggregation and recombination center formation. The sensing capability of the heterostructures was demonstrated in Fig. 4B, where the 10% Bi/h-Bi_2_Te_3_ NS exhibited a dramatic photocurrent enhancement (from 1.94 to 2.81 µA cm^−2^) in the presence of 1 µM APAT, compared to the modest response of the pristine material (from 0.89 to 1.16 µA cm^−2^). This enhancement arose from the hole scavenger effect, where APAT molecules adsorbed onto the electrode surface and underwent preferential oxidation, suppressing electron–hole recombination. The 10% Bi/h-Bi_2_Te_3_ heterostructure further amplified the sensing signal through enhanced charge separation efficiency and prolonged carrier lifetime.

Electrochemical impedance spectroscopy (EIS) Nyquist plots confirmed the improved interfacial charge transfer dynamics, with the 10% Bi/h-Bi_2_Te_3_ NS displaying the smallest semicircle diameter (796.4 Ω) under illumination with 1 µM APAT, representing an 46.1% reduction compared to the pristine material (Fig. 4C). The optimal bias potential for photoelectrochemical response to 1 µM APAT was determined to be +0.2 V (Fig. 4D), where the photocurrent reached a maximum of 2.93 µA cm^−2^. A concentration-dependent analysis revealed a linear relationship (Δ*I* = 0.7142*C* + 0.2781, *R*^2^ = 0.9858) between photocurrent enhancement and APAT concentration from 0.5 to 10 µM, with a limit of detection (LOD) of 0.08 µM and a limit of quantification (LOQ) of 0.26 µM (Fig. 4E). The increased photocurrent can be attributed to the progressive adsorption and oxidation of apatinib molecules at the photoelectrode–electrolyte interface, where apatinib serves as an efficient hole-scavenging/electroactive species to suppress electron–hole recombination. Therefore, the linear response in Fig. 4E demonstrates the feasibility of quantitative apatinib detection using the Bi/h-Bi_2_Te_3_ PEC sensing platform. Long-term stability and reproducibility assessments (Fig. 4F) demonstrated that the 10% Bi/h-Bi_2_Te_3_ photoanode maintained 98.7% of its initial photocurrent after 1400 s of continuous operation with 1 µM APAT, indicating excellent operational stability. The developed Bi/h-Bi_2_Te_3_ PEC sensor delivers favorable apatinib detection performance with a low LOD and reliable sensitivity, offering a simpler and more stable sensing alternative to reported PEC platforms and conventional LC-MS/MS methods (Table S2). Selectivity evaluation against common biologically relevant species, including ascorbic acid (AA, 50 µM), uric acid (UA, 50 µM), glucose (Glu, 1 mM), and urea (1 mM) at 50–1000-fold excess confirmed that the sensor response remained essentially unaffected, with individual interference below 10% (Fig. S2), demonstrating excellent anti-interference performance.

**Fig. 5:**
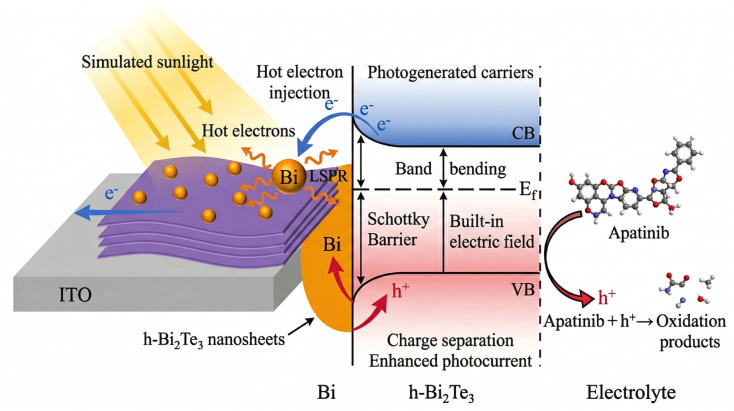
Schematic illustration of the proposed charge transfer mechanism in the photoelectrochemical (PEC) biosensing platform for APAT detection.

Fig. 5 schematically illustrates the integrated photoelectrochemical sensing mechanism in the ITO/Bi/h-Bi_2_Te_3_/electrolyte system for APAT detection. Under AM 1.5G simulated solar illumination, the system operates through a series of synergistic processes. Visible light absorption by h-Bi_2_Te_3_, with a bandgap of 1.78 eV, generates electron–hole pairs.^45^ Simultaneously, metallic Bi undergoes localized surface plasmon resonance (LSPR) excitation, enhancing near-field electromagnetic intensity and producing hot electrons. A Schottky barrier at the Bi–Bi_2_Te_3_ interface, creates a built-in electric field that drives photogenerated electrons from the conduction band (CB) of h-Bi_2_Te_3_ to metallic Bi, which serves as an electron sink due to its lower Fermi level.^46^ These accumulated electrons are then extracted to the external circuit *via* the ITO electrode, generating a measurable photocurrent. Meanwhile, photogenerated holes in the valence band (VB) of h-Bi_2_Te_3_ migrate to the electrode–electrolyte interface. Adsorbed APAT molecules undergo oxidation by these holes (apatinib + h^+^ → apatinib^+^˙ → electrooxidation products) with faster kinetics than competing water oxidation, effectively consuming holes and suppressing electron–hole recombination.^47,48^ This results in an enhanced photocurrent proportional to the APAT concentration, enabling quantitative detection. The synergistic effects of LSPR-enhanced light absorption, Schottky barrier-facilitated charge separation, efficient electron extraction *via* metallic Bi, and catalytic signal amplification through APAT oxidation collectively enable high-sensitivity and stable photoelectrochemical sensing.

### 3.5. Machine learning-based recurrence prediction for post-TACE HCC patients

**Fig. 6:**
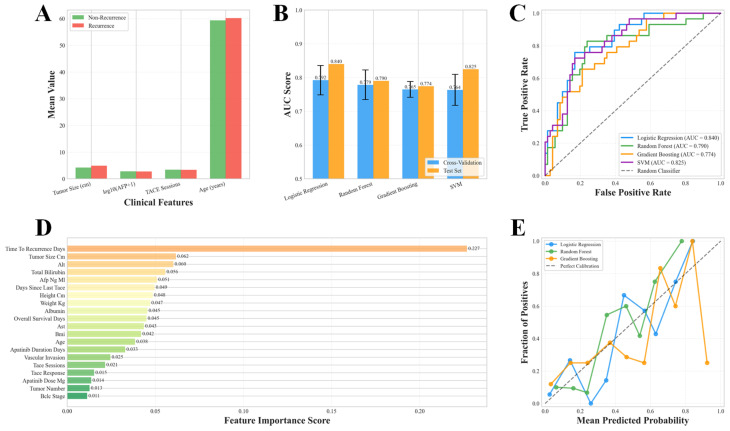
(A) Feature distribution by recurrence status. (B) Model performance comparison. (C) ROC curves for HCC recurrence prediction. (D) Top 20 feature importance ranking. (E) Calibration curves.

A total of 500 patients with hepatocellular carcinoma (HCC) who underwent transcatheter arterial chemoembolization (TACE) were included in this study (Table S3). The mean age was 59.6 ± 11.8 years, with 351 (70.2%) male patients, reflecting the typical demographic distribution of HCC. Among these patients, 145 (29.0%) experienced tumor recurrence during the follow-up period, with a median time to recurrence of 242 days (interquartile range: 95–512 days). Baseline characteristics were generally balanced between the recurrence and non-recurrence groups. However, patients with recurrence had significantly larger tumor sizes (4.90 ± 2.57 cm *vs.* 4.25 ± 2.46 cm, *p* = 0.023), higher rates of vascular invasion (39.3% *vs.* 26.8%, *p* = 0.012), and were more likely to have progressive disease as the best TACE response (20.0% *vs.* 12.4%, *p* < 0.001). The median alpha-fetoprotein (AFP) level was numerically higher in the recurrence group (55.6 ng mL^−1^*vs.* 45.7 ng mL^−1^), though this difference did not reach statistical significance (*p* = 0.234). Importantly, 261 (52.2%) patients received APAT therapy following TACE, with a mean dose of 375 ± 128 mg day^−1^ and a median treatment duration of 157 days (IQR: 72–298 days). Key clinical features, including tumor size, AFP level (log-transformed), TACE sessions, and age, demonstrated distinct distribution patterns between recurrence and non-recurrence groups, with tumor size showing the most pronounced difference (Fig. 6A). These baseline characteristics are consistent with previous reports on HCC patient populations undergoing TACE. The relatively high proportion of hepatitis B-positive patients (69.6%) reflect the epidemiological pattern in regions where hepatitis B virus (HBV) is endemic. The predominance of Child-Pugh A classification (65.4%) indicates appropriate patient selection for TACE therapy, as recommended by current guidelines. Four machine learning models were developed and validated for predicting HCC recurrence (Table S4). Among these models, logistic regression demonstrated the best overall performance with an area under the receiver operating characteristic curve (AUC) of 0.840 (95% confidence interval: 0.776–0.904), accuracy of 78.0%, sensitivity of 72.4%, and specificity of 80.3% on the independent test set. Cross-validation analysis confirmed the stability and generalizability of model performance, with mean cross-validation AUC ranging from 0.764 to 0.792 across all models (Fig. 6B). The support vector machine model showed comparable performance with an AUC of 0.825 (95% CI: 0.759–0.891), while ensemble methods, including random forest (AUC = 0.790) and gradient boosting (AUC = 0.774), yielded moderate predictive ability (Fig. 6C). The close agreement between cross-validation and test set performance (difference < 0.05 for all models) suggests minimal overfitting and good model generalization. The logistic regression model achieved a cross-validation AUC of 0.792 ± 0.043, demonstrating consistent performance across different data subsets. The superior performance of logistic regression, despite its relative simplicity compared to ensemble methods, can be attributed to several factors, including well-normalized features, relatively linear relationships with the outcome, and the sample size (*n* = 500), which may be insufficient to fully leverage the capacity of more complex ensemble methods. Additionally, the feature importance analysis revealed that tumor size, alanine aminotransferase (ALT), total bilirubin, and AFP were among the most important predictors of HCC recurrence (Fig. 6D and Table S5). The logistic regression's probabilistic output is naturally well-calibrated, as evidenced by the calibration curves (Fig. 6E).

The prominence of tumor size aligns with established clinical knowledge that larger tumors are associated with higher recurrence risk due to increased tumor burden and higher likelihood of microscopic vascular invasion. The importance of liver function parameters highlights the critical role of liver reserve in determining treatment outcomes. AFP, the most widely used biomarker for HCC, confirmed its established prognostic value. Treatment-related features, including days since last TACE and APAT duration, also appeared in the top predictors, suggesting that treatment timing and duration significantly influence recurrence risk. Model calibration analysis demonstrated good agreement between predicted probabilities and observed recurrence rates (Fig. 6E). The logistic regression model showed the best calibration, with predicted probabilities closely tracking the diagonal line representing perfect calibration. This excellent calibration performance is particularly important for clinical application, ensuring that predicted probabilities align with actual outcomes. APAT therapy demonstrated significant benefits in reducing recurrence rates and improving survival outcomes (Table S6). The clinical link between apatinib and recurrence should be interpreted through therapeutic exposure rather than as a direct tumor-derived biomarker. In the post-TACE setting, local ischemia and hypoxia may activate HIF-1α/VEGF-driven angiogenesis in residual tumor tissue, which provides a biological basis for recurrence after embolization.^23^ Inadequate apatinib exposure may therefore reduce VEGFR-2 blockade and permit angiogenesis-driven regrowth, whereas excessive exposure may increase toxicity and lead to dose reduction or treatment interruption. Patients who received APAT had substantially lower recurrence rates (21.1% *vs.* 37.7%, *p* = 0.002) and longer median overall survival (815 days *vs.* 607 days, *p* = 0.012). However, adverse events were more common in the APAT group, with grade ≥ 3 adverse events occurring in 20.3% of APAT-treated patients compared to 2.1% in the control group (*p* < 0.001). Based on our findings, we propose a risk-stratified approach to apatinib therapy following TACE, guided by machine learning predictions, to optimize the benefit-risk ratio and improve resource allocation in clinical practice. It should be noted that model validation in this study was based on an internal hold-out test set from the same single-centre retrospective cohort, rather than an external or multi-centre validation dataset. Although stratified splitting and 5-fold cross-validation were used to reduce overfitting and assess internal robustness, the generalizability of the model beyond the present cohort remains to be confirmed. Future studies should validate using independent multi-centre datasets before clinical implementation.

## 4 Conclusion

In summary, this study successfully developed an integrated platform combining a high-sensitivity photoelectric sensor based on Bi/h-Bi_2_Te_3_ heterostructures with machine learning models for the detection and prediction of HCC recurrence following TACE. The Bi/h-Bi_2_Te_3_ heterostructures demonstrated exceptional photoelectrochemical performance, achieving a limit of detection of 0.08 µM for apatinib, a key therapeutic agent in HCC management. This was attributed to the synergistic effects of plasmonic enhancement, Schottky-junction facilitated charge separation, and efficient electron extraction. The stability and reproducibility of the sensor were validated over extended periods, ensuring reliable performance in practical applications. Complementing the sensor, machine learning models were developed to predict HCC recurrence using comprehensive clinical data from 500 patients. The logistic regression model exhibited superior performance with an area under the receiver operating characteristic curve (AUC) of 0.840, accuracy of 78.0%, sensitivity of 72.4%, and specificity of 80.3% on the independent test set. This highlights the potential of integrating sensor data with clinical variables to enhance predictive analytics in oncology. The integration of advanced materials engineering with clinical informatics represents a significant step toward precision medicine in HCC management.

## Conflicts of interest

There is no conflict of interest to declare by all the authors.

## Supplementary Material

RA-OLF-D6RA01947A-s001

## Data Availability

The data supporting this article have been included as part of the supplementary information (SI). Supplementary information is available. See DOI: https://doi.org/10.1039/d6ra01947a.
