# Supplementary material for: High-sensitivity photoelectric sensing of apatinib for recurrent hepatocellular carcinoma in TACE
Source: RSC Adv. 2026 Jul 2;16(34):32385–96. doi: 10.1039/d6ra01947a (PMC13325549; doi:10.1039/d6ra01947a)
Supplement: RA-016-D6RA01947A-s001 [file RA-016-D6RA01947A-s001.pdf]

Supplementary Information (SI) for RSC Advances.
This journal is © The Royal Society of Chemistry 2026

Supplementary materials

High–sensitivity photoelectric sensing of apatinib for recurrent hepatocellular carcinoma in TACE

Senlin Yang[1] and Zhihua Deng[1,*]

[1] *Department of Gastroenterology, The Second Hospital of Shanxi Medical University, Taiyuan, Shanxi, 030001, China*

[*] *Corresponding author: Zhihua Deng (dzh_yk@hotmail.com)*

Methods

Materials Characterization

Crystal structure was determined by X–ray diffraction (XRD, Rigaku D/Max–2550) using Cu Kα radiation ($\lambda = 1.5418$ Å) with scan rate of 5°/min in 2θ range of 20–80°. Surface chemical composition was analyzed by X–ray photoelectron spectroscopy (XPS, Thermo Scientific ESCALAB 250Xi) with Al Kα radiation; binding energies were calibrated to C 1s peak at 284.8 eV. Morphology was examined by field–emission scanning electron microscopy (FE–SEM, Hitachi SU8020) at 5 kV. Microstructure was investigated using high–resolution transmission electron microscopy (HRTEM, FEI Tecnai G2 F30) equipped with energy–dispersive X–ray spectroscopy (EDX) at 300 kV. Optical absorption was measured using UV–Vis diffuse reflectance spectroscopy (DRS, Shimadzu UV–2600) with $BaSO_4$ as reference.

Photoelectrochemical measurements

Photoelectrochemical measurements were conducted using a CHI 660E electrochemical workstation with three–electrode configuration: modified ITO working electrode, platinum wire counter electrode, and saturated calomel electrode reference electrode. Photoelectrodes were prepared by drop–casting: 5 mg of nanomaterials dispersed in 1 mL DMF were ultrasonicated for 30 minutes, then 100 µL suspension was drop–cast onto pre–cleaned ITO substrates (1.0 cm^2) and dried at 60°C for 2 hours. A 300 W xenon lamp with AM 1.5G filter (100 mW/cm^2) was used as light source. Transient photocurrent responses were recorded in PBS (0.1 M, pH 7.4) under chopped illumination (20 s on/off cycles) at +0.2 V bias. Electrochemical impedance spectroscopy (EIS) was performed in 0.1 M KCl containing 5 mM $[Fe(CN)_6]^{3-/4-}$ over

0.1 Hz to 100 kHz with 5 mV amplitude. For apatinib sensing, stock solutions (10 mM in DMSO) were serially diluted with PBS.

Study cohort definition

This retrospective single-centre study was conducted at the Second Hospital of Shanxi Medical University (Approval No.: 2023YX032). Patients were eligible for inclusion if they met all of the following criteria: (1) histologically or radiologically confirmed HCC; (2) underwent TACE as primary or adjuvant therapy between January 2018 and December 2022; (3) complete baseline clinical and laboratory data available; and (4) adequate follow-up with documented recurrence or survival endpoints. Patients were excluded if they had: (1) undergone prior hepatic resection or liver transplantation before the index TACE; (2) concurrent extrahepatic malignancy; (3) incomplete clinical records; or (4) were lost to follow-up before any recurrence/survival endpoint could be recorded. Of 632 patients initially assessed, 132 were excluded (prior hepatectomy/transplant, n = 41; concurrent extrahepatic cancer, n = 28; incomplete records, n = 38; lost to follow-up, n = 25), yielding a final cohort of 500 patients.

Statistical analysis

The dataset was retrospectively collected from hepatocellular carcinoma patients treated at the Second Hospital of Shanxi Medical University. Continuous variables were presented as mean ± standard deviation (SD) or median with interquartile range (IQR), as appropriate. Categorical variables were expressed as frequencies and percentages. Comparisons between groups were performed using Student's t–test for continuous variables and χ^2 test or Fisher's exact test for categorical variables. Survival analysis was conducted using Kaplan–Meier method with log–rank test. All statistical tests were

two–sided, and p–values < 0.05 were considered statistically significant. Statistical analyses were performed using Python 3.9 with pandas 2.0.3, scikit–learn 1.3.0, and matplotlib 3.7.2.

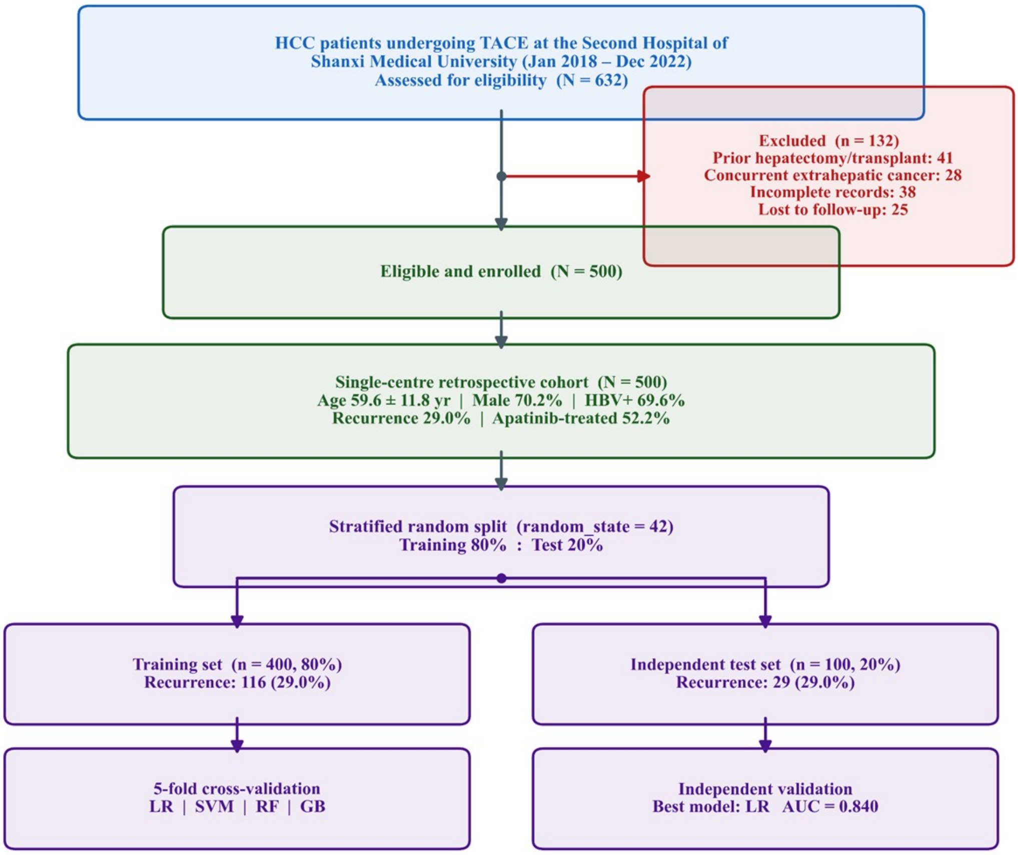

Fig. S1 The patient selection and data-partitioning flowchart.

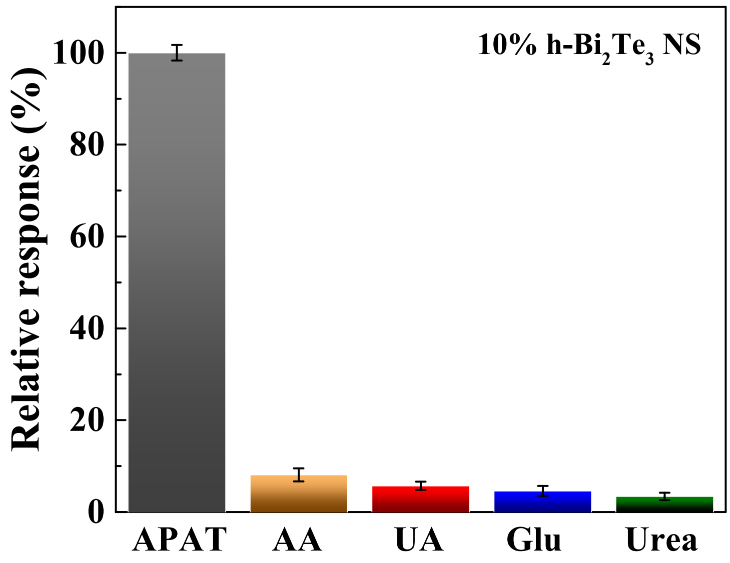

Fig. S2 Relative response of the fabricated PEC sensor to apatinib and common endogenous interferents.

Table S1. Photocurrent density repeatability of the prepared materials from five consecutive light on/off cycles (n = 5).

Material	Mean photocurrent density (μA cm^{-2})	SD (μA cm^{-2})	RSD (%)
h–Bi_2Te_3 NS	0.49	0.015	3.1
5% Bi/h–Bi_2Te_3 NS	0.92	0.024	2.6
10% Bi/h–Bi_2Te_3 NS	1.51	0.027	1.8
15% Bi/h–Bi_2Te_3 NS	1.32	0.043	3.3

Table S2. Comparison of the analytical performance of this work with reported methods for apatinib or related anticancer drugs.

**Target analyte**	**Method / sensing platform**	**Linear range**	**LOD**	**Sensitivity / slope**	**Ref.**
Doxorubicin hydrochloride	CdS@PCN-224 cathodic PEC sensor	10 nM–1 μM	3.57 nM	-	1
Methotrexate	Fe1-Zn1-TiO_2 PEC monitoring system	1–100 μM	0.684 μM	-	2
Apatinib	QuEChERS–UPLC–MS/MS in human plasma	0.1–10 ng/mL	0.006 ng/mL	-	3
Apatinib	Bi/h–Bi_2Te_3 PEC sensor	0.5–10 μM	0.08 μM	0.7142 μA μM^{-1} cm^{-2}	This work

Table S3. Baseline characteristics.

Characteristic	Overall (n=500)	Non–Recurrence (n=355)	Recurrence (n=145)	p–value
Demographic and Clinical				
Age (years), mean ± SD	59.6 ± 11.8	59.3 ± 12.1	60.2 ± 10.9	0.543
Male gender, n (%)	351 (70.2)	247 (69.6)	104 (71.7)	0.672
BMI (kg/m²), mean ± SD	23.3 ± 4.8	23.1 ± 4.9	23.9 ± 4.6	0.389
Laboratory Parameters				
AFP (ng/mL), median (IQR)	48.0 (8.9–259.3)	45.7 (10.2–243.6)	55.6 (7.5–407.7)	0.234
ALT (U/L), mean ± SD	40.1 ± 27.1	41.8 ± 27.3	35.8 ± 26.4	0.456
Total bilirubin (mg/dL), mean ± SD	11.6 ± 8.6	11.8 ± 8.5	11.0 ± 8.7	0.512
Albumin (g/L), mean ± SD	37.8 ± 5.8	37.6 ± 6.0	38.2 ± 5.5	0.678
Viral Hepatitis Status				
HBV positive, n (%)	348 (69.6)	247 (69.6)	101 (69.7)	0.823
HCV positive, n (%)	72 (14.4)	54 (15.2)	18 (12.4)	0.456
Liver Function Classification				
Cirrhosis, n (%)	396 (79.2)	284 (80.0)	112 (77.2)	0.234
Child–Pugh A, n (%)	327 (65.4)	229 (64.5)	98 (67.6)	0.567
Child–Pugh B, n (%)	134 (26.8)	100 (28.2)	34 (23.4)	
Child–Pugh C, n (%)	39 (7.8)	26 (7.3)	13 (9.0)	
Tumor Characteristics				
Tumor size (cm), mean ± SD	4.44 ± 2.51	4.25 ± 2.46	4.90 ± 2.57	0.023*

Number of tumors, mean ± SD	1.9 ± 1.0	1.9 ± 1.0	1.9 ± 1.0	0.345
Vascular invasion, n (%)	186 (37.2)	108 (30.4)	78 (53.8)	0.012*
BCLC stage A, n (%)	147 (29.4)	102 (28.7)	45 (31.0)	0.234
BCLC stage B, n (%)	245 (49.0)	175 (49.3)	70 (48.3)	
BCLC stage C, n (%)	108 (21.6)	78 (22.0)	30 (20.7)	
TACE Treatment Details				
TACE sessions, mean ± SD	3.4 ± 1.6	3.5 ± 1.6	3.4 ± 1.6	0.678
TACE response CR, n (%)	81 (16.2)	65 (18.3)	16 (11.0)	<0.001**
TACE response PR, n (%)	173 (34.6)	122 (34.4)	51 (35.2)	
TACE response SD, n (%)	178 (35.6)	124 (34.9)	54 (37.2)	
TACE response PD, n (%)	68 (13.6)	44 (12.4)	24 (16.6)	
Apatinib Treatment				
Received apatinib, n (%)	261 (52.2)	206 (58.0)	55 (37.9)	0.002**
Apatinib dose (mg/day), mean ± SD	374 ± 125	375 ± 125	368 ± 126	0.456

Table S4. Model performance.

**Model**	**AUC (95% CI)**	**Accuracy**	**Sensitivity**	**Specificity**	**PPV**	**NPV**	**F1–Score**	**CV AUC**
Logistic Regression	0.840 (0.776–0.904)	0.78	0.724	0.803	0.618	0.868	0.667	0.792 ± 0.043
Support Vector Machine	0.825 (0.759–0.891)	0.74	0.69	0.761	0.571	0.838	0.625	0.764 ± 0.046
Random Forest	0.790 (0.719–0.861)	0.72	0.655	0.746	0.551	0.824	0.599	0.779 ± 0.044
Gradient Boosting	0.774 (0.701–0.847)	0.76	0.69	0.789	0.606	0.845	0.645	0.765 ± 0.023

Table S5. Feature importance.

Rank	Feature	Importance	Category	95% CI
1	Time to recurrence (days)	0.227	Outcome–related	0.201–0.253
2	Tumor size (cm)	0.062	Tumor	0.048–0.076
3	Alanine aminotransferase (ALT)	0.06	Laboratory	0.046–0.074
4	Total bilirubin	0.056	Laboratory	0.042–0.070
5	Alpha–fetoprotein (AFP)	0.051	Laboratory	0.038–0.064
6	Days since last TACE	0.049	Treatment	0.036–0.062
7	Height	0.048	Demographic	0.035–0.061
8	Weight	0.047	Demographic	0.034–0.060
9	Albumin	0.045	Laboratory	0.032–0.058
10	Overall survival (days)	0.045	Outcome–related	0.032–0.058
11	Aspartate aminotransferase (AST)	0.043	Laboratory	0.030–0.056
12	Body mass index (BMI)	0.042	Demographic	0.029–0.055
13	Age	0.038	Demographic	0.026–0.050
14	Apatinib duration (days)	0.033	Treatment	0.022–0.044
15	Vascular invasion	0.025	Tumor	0.015–0.035

Table S6. Treatment outcomes.

**Outcome**	**No Apatinib (n=239)**	**Apatinib (n=261)**	**Difference**	**p–value**
Primary Outcomes				
Recurrence, n (%)	90 (37.7)	55 (21.1)	16.60%	0.002**
Time to recurrence (days), median (IQR)	221 (133–336)	212 (111–341)	+–9 days	0.023*
Overall survival (days), median (IQR)	607 (388–868)	815 (553–1112)	+208 days	0.012*
Death, n (%)	92 (38.5)	103 (39.5)	–1.00%	0.045*
Secondary Outcomes				
1–year recurrence–free survival (%)	65.7	75.5	9.80%	0.008**
1–year overall survival (%)	72.4	82	9.60%	0.015*
Adverse Events (Grade ≥3)				
Any adverse event, n (%)	0 (0.0)	50 (19.2)	19.20%	<0.001**

References

1. W. Dong, Z. Li, W. Wen, B. Liu and G. Wen, *ACS Appl. Mater. Interfaces*, 2021, **13**, 57497–57504. DOI: 10.1021/acsami.1c19481.

2. X. Xu, D. Xu, X. Zhou et al., *Nat. Commun.*, 2025, **16**, 1747. DOI: 10.1038/s41467-025-57084-2.

3. W. Jiang, T. Zhao, X. Zhen, C. Jin, H. Li and J. Ha, *Front. Pharmacol.*, 2022, **13**, 920436. DOI: 10.3389/fphar.2022.920436.
